# Concomitant ALK Fusion and TP53/EGFR Mutation Lead to Adverse Prognostic Outcome

**DOI:** 10.1111/crj.70041

**Published:** 2024-12-16

**Authors:** Mingyuan Du, Cuiwei Liu, Leichong Chen, Zhenyu Li, Sijia Zhang, Rui Meng

**Affiliations:** ^1^ Cancer Center, Union Hospital, Tongji Medical College Huazhong University of Science and Technology Wuhan China; ^2^ Hubei Key Laboratory of Precision Radiation Oncology Wuhan China; ^3^ Institute of Radiation Oncology, Union Hospital, Tongji Medical College Huazhong University of Science and Technology Wuhan China

**Keywords:** ALK fusion, concomitant, EGFR mutation, NSCLC, TP53 mutation

## Abstract

Lung cancer treatment has evolved at the molecular level. Detecting the presence of driver genes in lung cancer fundamentally alters the choice of therapeutic regimens and the outcome of this disease. ALK fusion mutation is one of the most important mutations in nonsmall cell lung cancer (NSCLC). Also, it often has other coexisting mutation types. TP53 is the most common coexisting mutation type, whereas the EGFR/ALK coexisting mutation type is extremely rare. There is still no definite conclusion about the impact of the multimutation and best treatment options for NSCLC patients with advanced multimutation. In this study, we report three cases of NSCLC with ALK fusion mutations as well as ALK combined with TP53 mutations and ALK combined with EGFR mutations. Combining cases from our oncology center and previous literature, we found that NSCLC patients with coexisting ALK fusion mutations and other mutations have poorer response to targeted therapy and poorer prognosis, and we also compared the efficacy rates of various types of coexisting mutations for different treatment regimens. Therefore, this review can help to evaluate the prognosis of NSCLC patients with coexisting mutations and the efficacy of targeted therapies and to find more favorable treatment options for patients with this type of coexisting mutations.

## Graphical abstract

This article reports three cases of nonsmall cell lung cancer with ALK fusion mutation, ALK combined with TP53 mutation, and ALK combined with EGFR mutation. It also compares the epidemiological characteristics of these three types of ALK coexisting mutations, evaluates the efficacy of chemotherapy and targeted therapy, and explores the mechanisms of resistance to targeted therapy, aiming to identify more effective treatment strategies for patients.

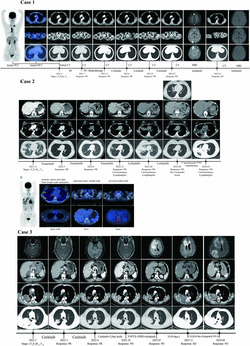

## 1 Introduction

Lung cancer is currently the cancer with the highest incidence rate worldwide [1, 2]. Nonsmall cell lung cancer (NSCLC), which accounts for more than 85% of total lung cancer, is the most predominant pathological type [3, 4]. NSCLC is recognized as a highly complex and heterogeneous disease [5, 6]. Recently, research related to genetic mutations and genomic heterogeneity has progressed rapidly, and quite a considerable number of driver genes in NSCLC have been discovered. The current types of driver gene in NSCLC can be roughly classified into “druggable mutation” and “undruggable mutation” based on the availability of effective targeted drugs [7]. ALK fusion mutation is one of the most important types of druggable mutations [8]. We found that the ALK fusion mutation could coexist with the most predominant druggable mutation EGFR and the most predominant undruggable mutation TP53. Although drugs targeted to the driver genes can significantly improve patients' prognosis, there is not a definite conclusion about the best treatment for patients with ALK fusion mutations combined with other types of mutations and the impact of concomitant mutations on the survival of NSCLC patients.

We presented three cases in our cancer center to analyzed the similarities and differences of NSCLC patients with ALK fusion mutation combined with or without concomitant mutations in Table 1. And we make a brief comparison of epidemiology, clinical features, drug response, and prognosis of ALK+ patients between those concomitant with TP53 mutation or EGFR mutation in Table 2.

**TABLE 1:** Clinicopathological features of the three cases.

	Case 1	Case 2	Case 3
Sex	Female	Female	Male
Age	49	45	59
Smoking	Never	Never	Never
Pathology	LADC	LADC	LADC
Mutation	EGFR missence mutation (E18) + EML4 (E6)‐ALK (E20) + TP53 mutation	EML4 (E20)‐ALK (E20) + TP53 mutation	HADHA (E4)‐ALK (E19)
Initial TNM Stage	IIIB (cT1N3M0)	IVB (cT4N3M1c)	IVB (cT4N3M1c)
Metastasis	—	Lung, bone, liver	Lung, bilateral adrenal, brain, bone
ECOG	0	2	0
Initial treatment	PC*3 + P*1 + RT Ceritinib	Ensartinib	Ceritinib RT (brain lesion)
Initial response	PR	PR	PR
Initial mPFS	22 months	13 months	8 months

Abbreviations: ECOG, Eastern Cooperative Oncology Group; LADC, lung adenocarcinoma; mPFS, median progression‐free survival; P, pemetrexed; PC, pemetrexed and carboplatin; PR, partial response; RT, radiotherapy.

**TABLE 2:** Comparison of epidemiological characteristics, clinical features, treatment, and prognosis of NSCLC patients with ALK/ALK + EGFR/ALK + TP53 mutations.

		ALK	ALK + EGFR	ALK + TP53
Incidence		3%–13%^a^ [9, 10]	0.3–1.3%^b^ [9, 11]	23.4%–60%^b^ [12, 13]
Age		Younger patients	Younger patients	Younger patients
Sex		No gender preference	Female	No gender preference
Ethnicity		Asian	Asian	Asian
Smoking		Never	Never	Are or have been
Pathology		LADC	LADC	LADC
Stage		advanced stage	advanced stage	advanced stage
Response to chemotherapy^c^		similar with ALK—	similar with ALK+	poorer than ALK+
Response to ALK‐TKIs^d^	ORR	81.6% [14], 73.9% [15], 65% [16], 60%–75.5% [17, 18, 19], 76.9% [20]	40% [15], 66.7% [16]	40% [14], 37.5% [20]
	DCR	95.9% [14]	—	73.3% [14]
mPFS	Chemotherapy	6.2m [13, 21] 7m [22], 8.1m [23],	—	2.6m [13, 21]
	ALK‐TKIs	29.9m [21], 6.9m [15], 11.7m [24], 12.5‐13 m [16, 25], 9.3‐11 m [17, 18, 19], 27.9m [26], 10.4–28.5 [27] 8.8 [28] 10.8 [29]	1.9m [15], 11.1m [16] 2m [30]	5.5m [21], 4.2m [24], 8m [25], 3.3m [20], 9.2m [26] 3.7 [28] 7.2 [29]
	EGFR‐TKIs	—	11.2m [9, 15], 10.3m [16]	—
mOS		50m [13], 23.7m [15], 34.2m [20],	18.5m [15]	15m [13], 21.4m [20]

Abbreviations: DCR, disease control rate; LADC, lung adenocarcinoma; m, month; mOS, median overall survival; mPFS, median progression‐free survival; ORR, objective response rate.

^a^ In all nonsmall cell lung cancer patients.

^b^ In ALK fusion mutation nonsmall cell lung cancer patients.

^c^ Platinum‐based first‐line chemotherapy regimen.

^d^ ALK‐TKI represented by crizotinib.

## 2 Case Presentation

### 2.1 Case 1

**FIGURE 1:**
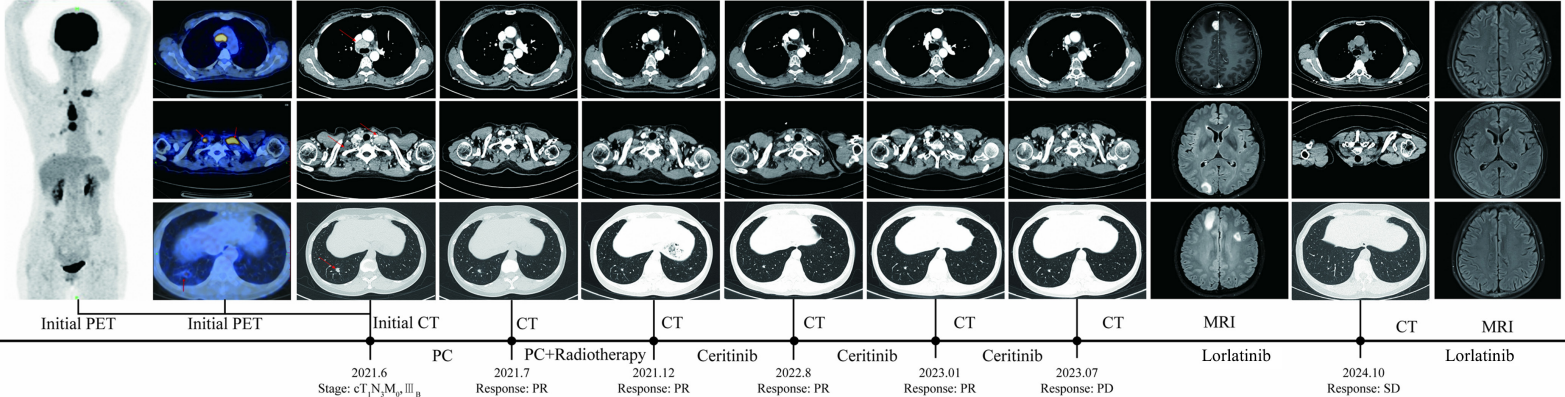
The initial PET/CT scans and CT scans before and after treatment of Case 1. PC: the first cycle of pemetrexed (500 mg/m^2^) and carboplatin (AUC: 5) chemotherapy; PC + Radiotherapy:pemetrexed (500 mg/m^2^) and carboplatin (AUC: 5) every 3 weeks for the following two cycles and pemetrexed (500 mg/m^2^) for the fourth cycle, with concurrent supraclavicular and mediastinal region radiotherapy (PTV:60Gy/30F and GTVnd:66Gy/30F). Ceritinib: 450 mg orally per day. Lorlatinib: 100 mg orally per day; CT, computed tomography; MRI, nuclear magnetic resonance imaging; PD, progressive disease; PET, positron emission tomography; PR, partial remission; SD, stable disease. The red arrows point to the primary lung cancer and metastatic lymph nodes.

A 49‐year‐old female patient with no history of smoking presented to the hospital because of enlarged left supraclavicular lymph nodes in June 2021. Positron emission tomography/computed tomography (PET/CT) showed a nodule in the posterior basal segment of right lower lung lobe with multiple enlarged lymph nodes in supraclavicular fossa (Figure 1). Then, supraclavicular lymph node puncture biopsy was performed in June 2021. The pathology results showed metastatic lung adenocarcinoma. Next‐generation sequencing (NGS) analysis displayed an ALK rearrangement EML4(E6)‐ALK(E20) with TP53 mutation (c.844C > T, p.Arg282Trp[E8]) and EGFR missense mutation (c.2161G > A, p.Gly721Ser[E18]). The patient was diagnosed with lung adenocarcinoma (cT1N3M0, Stage IIIB) with an ECOG (Eastern Cooperative Oncology Group) score of 0. After diagnosis, the patient received PC chemotherapy (pemetrexed: 500 mg/m^2^ + carboplatin: AUC 5) and radiotherapy (supraclavicular and mediastinal region, PTV: 60Gy/30F, GTVnd: 66Gy/30F) since June 2021. The efficacy was evaluated as partial remission (PR) after concurrent radiochemotherapy. Then, the patient was treated with ceritinib (450 mg per day), and the efficacy was evaluated as continuous PR until May 2023. In June 2023, magnetic resonance imaging (MRI) suggested the patient had several new brain metastases. Therefore, the patient was treated with lorlatinib instead of ceritinib. The patient is currently continuing with targeted therapy using lorlatinib, and the follow‐up examination in November 2024 indicates stable disease (SD).

### 2.2 Case 2

**FIGURE 2:**
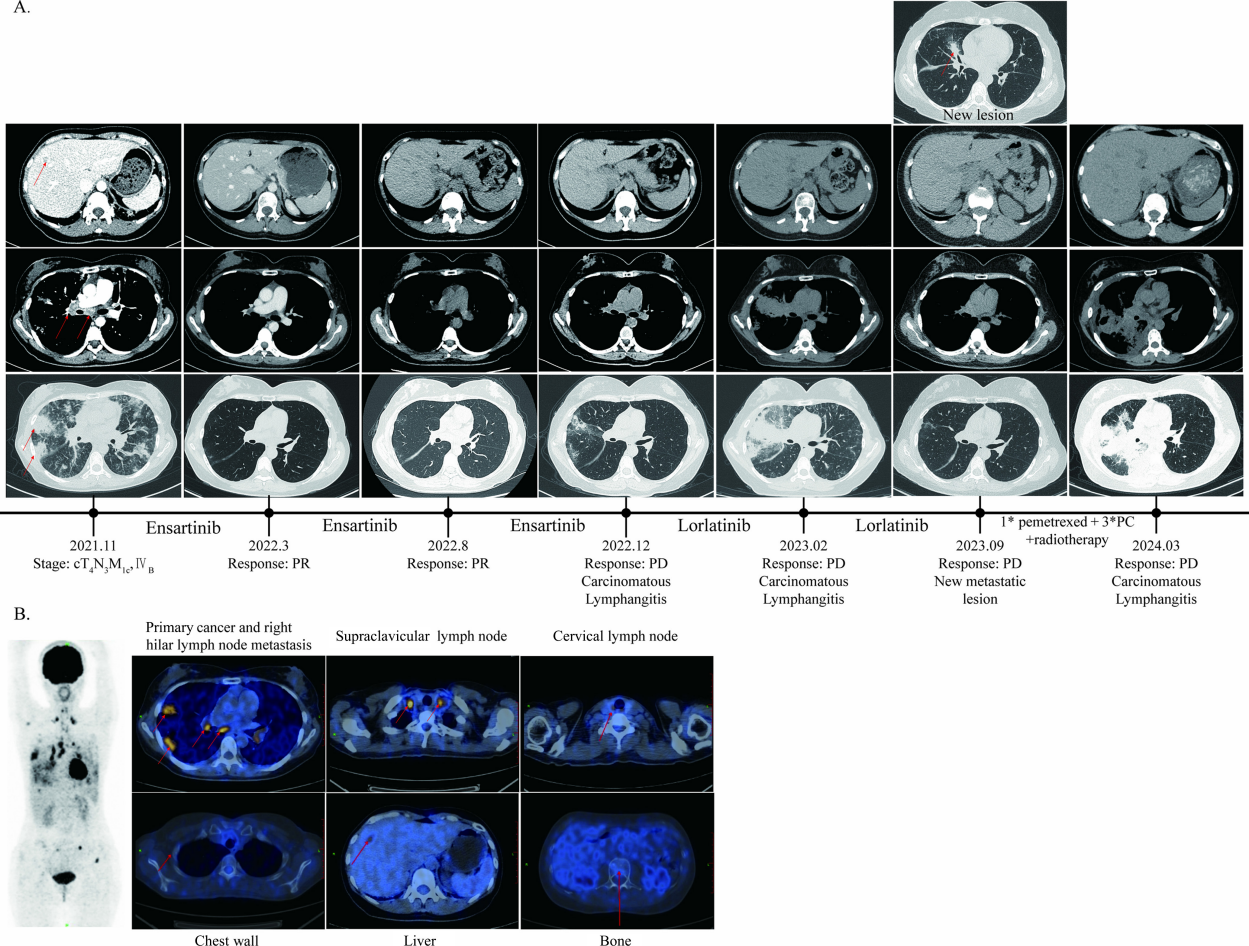
The initial PET/CT scans and CT scans before and after treatment of Case 2. (A) CT scans of Case 2 before and after treatment. Ensartinib: 225 mg orally per day; PC, pemetrexed + carboplatinum; PD, progressive disease; PR, partial remission. (B) Initial PET/CT scans of Case 2. The red arrows point to the primary lung cancer, metastatic lymph node, and metastatic lesions. Lorlatinib: 100 mg orally per day.

A 45‐year‐old female patient presented to our hospital in November 2021 due to 1 month of cough and a few days of dyspnea. CT scans and 18F‐FDG PET/CT showed a mass in the basal segment of lower lobe of right lung associated with multiple intrapulmonary and lymph node metastases, as well as the liver and bone metastases (Figure 2). The biopsy of lung nodules was performed, and the pathology results showed invasive lung adenocarcinoma. NGS test revealed EML4(E20)‐ALK(E20) fusion with TP53 mutation (shift code mutation at chr17:7574033). The diagnosis of this patient was lung adenocarcinoma (cT4N3M1c, Stage IVB), and ECOG score was 2.

Since November 2021, the patient was treated with ensartinib, 225 mg per day. The dyspnea of the patient was significantly relieved. The response evaluation was persistent PR. In December 2022, the patient had progressed dyspnea; CT scans indicated the progression of the disease. Therefore, the patient was treated with lorlatinib instead of ensartinib. The response evaluation was PR after lorlatinib treatment, and the dyspnea was significantly relieved in June 2023. In September 2023, a follow‐up CT scan revealed a new metastatic lesion in the right middle lobe of the lung, suggesting disease progression. Later, the patient began one cycle of pemetrexed therapy and three cycles of PC (pemetrexed + carboplatin) chemotherapy regimen. Also, from December 27, the patient's right lung lesions and the thoracic spine were treated with radiotherapy. And the radiotherapy doses were PTV 40Gy/10F and PTV 30Gy/10F, respectively. After completing this cycle of treatment, the CT scan in February 2024 indicated further tumor progression, and the patient experienced shortness of breath and low SpO2 levels. In March 2024, as the shortness of breath worsened, the patient was admitted to the hospital. During hospitalization, a thoracentesis was performed to drain hemorrhagic pleural effusion. Based on the pathology results, pleural metastasis was considered. Subsequently, the patient's condition continued to deteriorate. The medical team provided best supportive care (BSC) for her, and clinical death was declared on 25 March 2024.

### 2.3 Case 3

**FIGURE 3:**
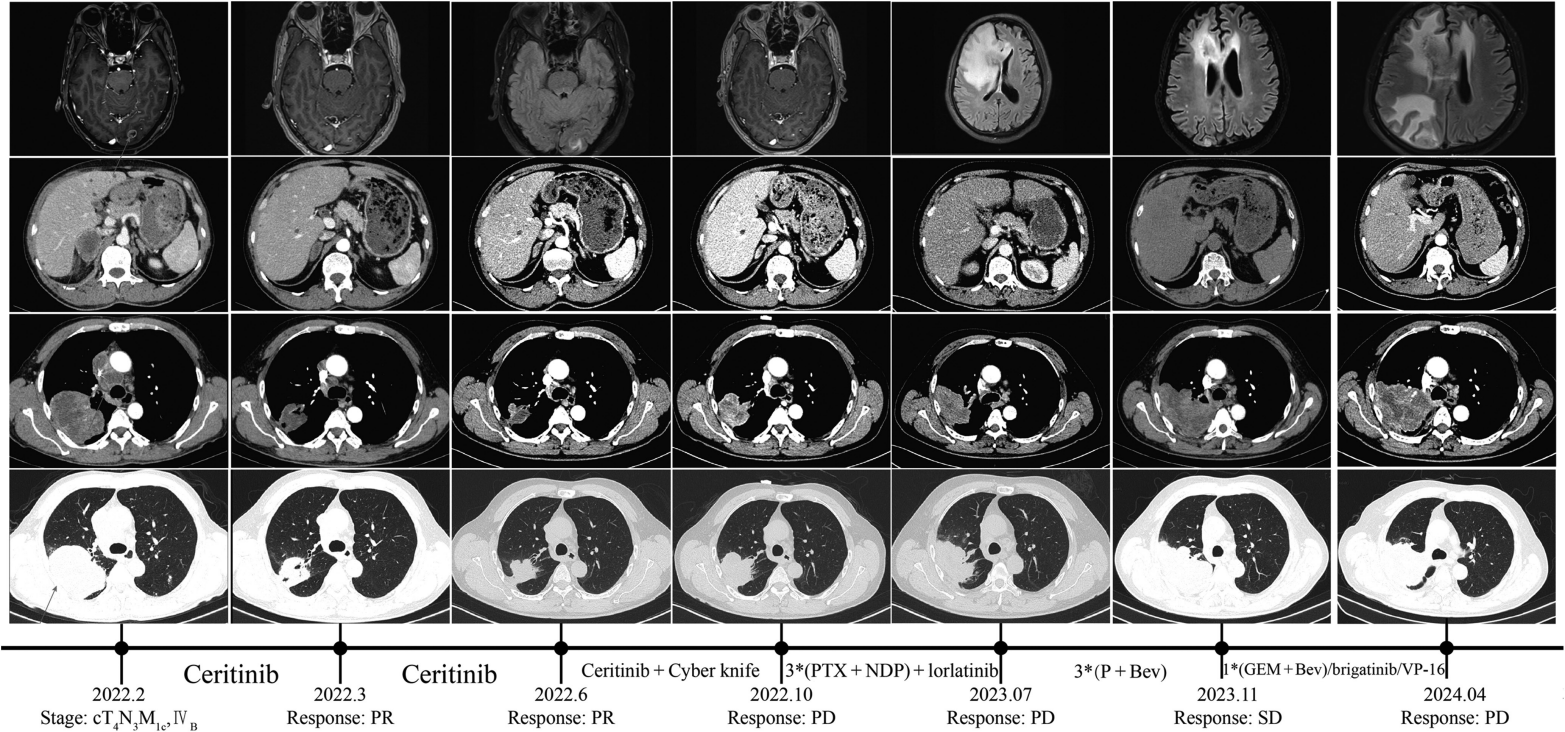
CT and MRI scans of Case 3 before and after treatment. Ceritinib: 450 mg orally per day; Ceritinib + Cyber knife: ceritinib 450 mg orally per day and brain lesion radiotherapy (dose: 33Gy/3F). Bev, bevacizumab; GEM, gemcitabine; NDP, nedaplatin; P, pemetrexed; PD, progressive disease; PR, partial remission; PTX, paclitaxel; SD, stable disease; VP16, etoposide. The red arrows point to primary lung cancer, metastatic lymph nodes, and metastatic lesions.

In February 2022, a 59‐year‐old male patient presented to the hospital because of intermittent cough and hemoptysis for more than 3 months. CT scans in February 2022 revealed a huge space‐occupying lesion in the right lung with multiple lymph nodes enlargement in mediastinum, as well as bilateral adrenal, brain, and bone metastasis (Figure 3). After CT‐guided lung puncture biopsy, the pathology results showed invasive lung adenocarcinoma. NGS test revealed HADHA(E4)‐ALK(E19) fusion mutation. The diagnosis of this patient was lung adenocarcinoma (cT4N3M1c, Stage IVB).

After diagnosis, the patient received ceritinib (450 mg per day) as the first‐line treatment since February 2022 and underwent radiotherapy to treat brain metastasis (CyberKnife, dose: 33Gy/3F) in July 2022. The efficacy was evaluated as persistent PR. However, the lung lesions of the patient progressed in October 2022. The patient subsequently underwent three cycles of chemotherapy with Paclitaxel combined with Nedaplatin regimen at local hospital; the lung lesions of the patient continued to progress. Therefore, the treatment of the patient was changed to lorlatinib since April 2023. On 16 July 2023, the patient experienced a sudden headache, and brain MRI indicated a new intracranial metastase. The patient then began three cycles of treatment with pemetrexed and bevacizumab. And the brain lesions were assessed as stable disease (SD), whereas the lung lesions were evaluated as disease progression (PD). Starting from 6 November 2023, the patient received one cycle of gemcitabine and bevacizumab. However, the treatment outcomes were still unsatisfactory. Consequently, treatment was switched to oral brigatinib. And the disease continued to progress, and then from February 2024, the patient was switched to oral chemotherapy with VP‐16(Etoposide). Despite these interventions, the disease continued to progress, and clinical death was declared on 12 May 2024.

## 3 Discussion

In this study, we presented three cases: Case 1 had EML4‐ALK fusion, concomitant with EGFR and TP53 mutation; Case 2 had EML4‐ALK fusion and TP53 mutation; and Case 3 only had HADHA‐ALK fusion. When ALK fusion mutation coexists with other mutations, especially druggable mutation, it is difficult to make clinical decisions and predict the prognosis of the NSCLC patients. EGFR is the most predominant druggable mutation, and TP53 is the most predominant undruggable mutation. Therefore, we collected the published literature and summarized the clinical characteristics, treatment response, and prognosis of the patients to help the clinicians understand this complex situation better.

### 3.1 ALK Fusion Mutations

#### 3.1.1 ALK Gene and ALK Fusion Mutations

The gene encoding ALK protein is located on the short arm of human chromosome 2 and was first identified in 1994 by Morris et al. [31]. ALK has a classical receptor tyrosine kinase (RTK) structure and belongs to the insulin receptor subfamily [32]. ALK protein can activate multiple signal pathways, which are highly involved in cell proliferation, differentiation, and antiapoptotic signaling processes [33].

ALK protein is not normally transcribed in adult lung [34, 35]. Our Cases 1 and 2 have EML4‐ALK fusion, whereas Case 3 have HADHA‐ALK, which is a rare translocation partner. Taking EML4‐ALK fusion as an example [36], the inversion of a portion of chromosome 2p juxtaposes the 5′ portion of EML4 to the 3′ portion of ALK. Under the control of EML4 promoter, the EML4‐ALK fusion gene is successfully transcribed [37]. The EML4‐ALK fusion protein is expressed in lung under the transcriptional control of EML4 promoter, and the dimerization domain of EML4 permits unregulated dimerization of the TK domain, persistently activating downstream pathways that lead to tumor formation [33, 37, 38].

#### 3.1.2 Clinical Utilization of ALK‐TKIs and Mechanisms of Drug Resistance

The ALK fusion mutation‐targeted drug ALK‐TKIs (ALK‐tyrosine kinase inhibitors), an ATP‐competitive small molecule inhibitor of tyrosine kinases, can significantly improve the prognosis of NSCLC patients with ALK fusion mutation, whose median progression‐free survival (mPFS) was improved from 7–8.1 months to 10.4–28.5 months [17, 22, 23, 27, 39, 40, 41, 42, 43, 44]. However, heterogeneous responses to ALK‐TKI have been reported. Different variants of ALK fusion, different translocation partners, and the presence of ALK point mutations may contribute to their heterogeneous responses to ALK‐TKI [7, 41]. In this study, the efficacy of ceritinib treatment in Case 3 was very limited; the PFS of Case 3 after ceritinib treatment and radiotherapy was only 8 months, which was obviously shorter than median PFS in clinical studies and might because of the rare translocation partner of ALK fusion mutation.

In previous published studies, the mechanisms of secondary resistance of ALK‐TKIs can be divided into ALK‐dependent and ALK‐independent. ALK secondary mutations are the most common reason for ALK‐dependent resistance. The ALK fusion protein structure and the change of responses to different ALK‐TKIs might due to the various secondary mutations. Fifty to sixty percent of patients received second‐generation ALK inhibitor acquire drug resistance because of the secondary ALK mutation [45, 46, 47]. Bypass signaling pathway activation is an important category for ALK‐independent resistance, which might because of genetic alterations, dysregulation of autocrine signaling or feedback signaling, resulting in reactivation of downstream pathway required for tumor cell growth and survival [48, 49, 50, 51, 52]. Multiple bypass signaling pathways have been demonstrated, including activation of RTKs: MET [53], EGFR [50], SRC [54], IGF‐1R [55], HER2 and HER3 [54], KIT [50], and alternation of their downstream signaling factors [27]. The recent studies also indicate that EML4‐ALK fusions drive lung adeno‐to‐squamous transition (AST) through JAK‐STAT activation, which further leads to ALK inhibitor resistance depend on the pathological transition [27, 56]. The polyclonal characteristic also contributes to the drug resistance, because the target therapy drugs only inhibit one specific type of cancer cell [27].

### 3.2 ALK Fusion Mutation Concomitant With EGFR/TP53 Mutations

#### 3.2.1 Overview of Coexistence of ALK Fusion Mutation and EGFR/TP53 Mutations

TP53 mutation is the most common coexisting mutation type, accounting for 23.4%–60% of NSCLC patients with ALK fusion mutation (ALK+) [12, 13, 29]. In the study of Kron et al. [13], the frequency of pathogenic TP53 mutations was 23.8%, whereas all other coalterations occurred rarely with frequencies no more than 3.6%, among ALK+ NSCLC patients. ALK+ NSCLC patients concomitant with EGFR mutations were rare, accounting for only 0.3%–7.53% of ALK+ patients [9, 11, 57]. Patients with EGFR mutation and ALK fusion mutation were first reported in 2008 [58], and then, subsequent cases were also reported to demonstrate this phenomenon. A brief comparison of epidemiology, clinical features, drug response, and prognosis of ALK+ patients between those concomitant with TP53 mutation or EGFR mutation is presented in Table 2.

#### 3.2.2 Epidemiological and Clinical Characteristics of Coexistence of ALK Fusion Mutation and EGFR/TP53 Mutations

The NSCLC patients with ALK fusion mutation can be divided into three groups: patients only had ALK fusion mutation (ALK+), patients had ALK fusion mutation coexisting with EGFR mutation (ALK + EGFR), and patients had ALK fusion mutation coexisting with TP53 mutation (ALK + TP53).

The epidemiological and clinicopathological characteristics of these three groups were broadly similar. All tend to be found in younger Asian patients and advanced clinical stage patients, which might because of the extensive gene testing in advanced stage patients and limited testing in early‐stage patients. The pathological type is mainly lung adenocarcinoma in all three groups [10, 11, 14]. However, in the studies by Wang and Zhong [11], ALK + EGFR is more common in female than males with a ratio of 2:1. The reason for the gender preference might be contributed by EGFR, which is more common in female patients. And both ALK+ and ALK + TP53 groups do not clearly show a significant gender preference [10, 11]. Furthermore, the ALK + TP53 group shows an association with smoking history, which is different with the ALK+ and ALK + EGFR groups. This difference might because of TP53 mutation, which is highly associated with smoking [14].

#### 3.2.3 Response to Chemotherapy of Coexistence of ALK Fusion Mutation and EGFR/TP53 Mutations

In platinum‐based combination chemotherapy, there was no significant difference in objective remission rate (ORR) between ALK+ and ALK− NSCLC patients [59]. A large number of studies have shown that concomitant with TP53 mutations is a poor response factor for patients with ALK fusion mutations, with shorter median progression‐free survival (mPFS) (2.6 vs. 6.2 months) and median overall survival (mOS) (2.0 vs. 9.0 months) when treated with first‐line chemotherapy compared with TP53 wildtype patients [13, 21]. In our study, the Case 1 patient had ALK fusion mutation concomitant with both EGFR and TP53 mutations; this patient also showed a good response to the chemotherapy.

#### 3.2.4 Response to Target Therapy of Coexistence of ALK Fusion Mutation and EGFR/TP53 Mutations

EGFR and ALK, the two most important NSCLC driver genes, are generally considered to be mutually exclusive. The occurrence of EGFR/ALK coalterations in NSCLC is rare or uncommon genetic alteration events, with an incidence of 0.3%–1.3% [9, 11]. In recent study, for the first‐line EGFR‐TKIs treatment, the ORR (Objective Response Rate) was 57.1%–64%, and DCR (Disease Control Rate) was 64%–82% in 28 EGFR/ALK coaltered patients [11]. Yang et al. [9] also reported that 10 patients harboring EGFR/ALK coalterations treated with the first‐generation EGFR‐TKI, the ORR was 80%, and the mPFS was 11.2 months. Also, Luo et al. showed that for the treatment of crizotinib, the ORR was 40% for EGFR/ALK coaltered and 73.9% for ALK− positive, with mPFS of 1.9 and 6.9 months, respectively [15]. The study included a small number of people with EGFR/ALK and ALK‐positive mutation using crizotinib, which may be one of the reasons for the lower mPFS, so large sample studies need to be conducted. And mOS for patients with EGFR mutations, ALK rearrangements, and EGFR/ALK coalterations was 21.3, 23.7, and 18.5 months, respectively [15]. The ORR to crizotinib was 65.0% for ALK positive patients and 66.7% for EGFR/ALK coaltered patients, with a mPFS of 12.5 and 11.1 months, respectively [16]. Also, for first‐line EGFR‐TKI treatment, the ORR was 63.2% for patients with EGFR/ALK comutations and 62.1% for patients with EGFR mutations with a median PFS of 10.3 and 11.4 months, respectively [16]. In a recent study reported, a patient with EGFR/ALK comutation showed only two‐month PFS [30].

Concomitant with TP53 was a poor effect factor in the response to ALK‐TKIs in patients with ALK fusion mutations [14, 28, 29]. In the study of Kron et al. [13], the mPFS of ALK positive and ALK + TP53 patients was 10.3 and 3.9 months, and the mOS was 50 and 15 months, respectively. Reports show that the ORR for ALK/TP53 coalterations patients and ALK fusion patients treated with crizotinib were 40% versus 81.6%, and DCR was 73.3% versus 95.9% [14]. In a multicenter Phase 2 trial, Ensatinib was used to treat ALK‐positive NSCLC patients whose disease had progressed after crizotinib treatment. Patients with TP53 mutations had significantly shorter PFS compared to patients with TP53 wild type (TP53 mutant group: 4.2 months compared to 11.7 months in the TP53 wild group) [24]. In the crizotinib‐alone cohort, patients with TP53 mutant had significantly shorter mPFS compared to noncarriers (mPFS: 8 vs. 13 months) [25]. TP53 mutations were linked to a threefold reduction in mPFS of 3.7 months compared to 10.8 months and mOS of 42.2 versus 88.9 in a German program [28]. In our Case 3, the patient was ALK‐positive with no coexisting mutations, but his PFS to ceritinib was short, probably as a result of a rare translocation partner and brain metastases. In contrast, our patient in Case 1 had two concurrent mutations, but she still responded well to targeted therapy. The relatively early clinical staging and concurrent radiation chemotherapy may explain the better prognosis of the patient in Case 3.

### 3.3 Generation of Concomitant Mutations and Mechanisms of Drug Resistance

#### 3.3.1 Generation of Concomitant With EGFR Mutations and Mechanisms of Drug Resistance

In previous studies, there are two main hypotheses to explain the generation of concomitant mutations: (1) tumor heterogeneity, different tumor cells carry different tumor‐driver genes, and tumors may consist of two or more than two types of cancer cells, which carrying EGFR mutations or ALK fusion mutations; (2) the same tumor cell carry two or more tumor‐driver genes, and tumors consist of one type of cancer cells, which carrying both EGFR mutations and ALK fusion mutations. Those are the polyclonal origin and monoclonal origin hypotheses [11]. Both hypotheses have been studied to prove their plausibility [60, 61].

The drug resistance in NSCLC patients with both ALK and EGFR mutations may be the result of multiple factors. From the perspective of polyclonal origin hypothesis, it maybe relates to the alterations in the proportion of different types of tumor cells after target therapy, which might only inhibit the growth of one type of tumor cells. Thus, it lead to the change in the dominant type of tumor cells and finally the tumors show resistance to the target agents as a whole. Whereas, from the perspective of monoclonal origin hypothesis, it maybe relates to the bypass pathway activation [15, 50]. Whether based on the polyclonal or monoclonal origin hypothesis, the treatment of EGFR‐TKIs and ALK‐TKIs in combination is the better choice for patients had ALK fusion concomitant with EGFR mutations. However, whether the combination treatment will improve the prognosis of patients is unknown. Some researchers suggest that the choice of EGFR‐TKIs or ALK‐TKIs should be based on the mutation abundance and relative phosphorylation levels of the corresponding EGFR and ALK [40, 58].

#### 3.3.2 Generation of Concomitant With TP53 Mutation and Mechanism of Drug Resistance

TP53 mutations were closely associated with enhanced chromosomal instability, including increased oncogenes amplification and deep deletion of suppressor genes [62] and occurred in early tumorigenesis [13, 63]. Most studies suggest that the mechanisms of ALK‐TKIs resistance are associated with chromosomal instability due to TP53 in NSCLC patients with both ALK and TP53 mutations. In the study by Alidousty et al. [63], 14% of NSCLC patients with both ALK and TP53 mutations have MYC amplification, which lead to EML4‐ALK upregulation and suggest a potential MYC‐dependent mechanism for ALK‐TKIs resistance. Increased MYC copy number might be the only one type of TP53‐related drug resistance. CCND1 (10%), TERT (5%), BIRC2 (5%), ORAOV1 (5%), YAP1 (5%), and other known oncogene amplifications are also detected in TP53 positive tumors [63], which might also associate with the drug resistance due to TP53 mutations. In the study of Tanimoto et al. [26], loss of normal p53 function results in resistance to ALK‐TKIs in ALK+ NSCLC and combined proteasome inhibitor with alectinib is a promising therapy for ALK+TP53 NSCLC.

## 4 Conclusion

With the popularization of the NGS tests, more and more NSCLC patients with concomitant mutations had been found. Current literature suggests that the coexistence of ALK fusion and other mutations might lead to alterations in epidemiological characteristics, clinical features, treatment options, drug response, prognosis, and survival of NSCLC patients. Till now, there is no definitive conclusion on the best treatment for NSCLC patients with ALK fusion combined with other mutations, especially druggable mutations. It is hard to identify whether the patient with druggable concomitant mutations can achieve better clinical outcomes by combining targeted agents. The concomitant mutations should be taken into account as an adverse factor in the selection of treatments and prognostic assessment. Further in‐depth clinical investigations are expected for developing better choices of treatments for NSCLC patients with concomitant mutations in addition to ALK fusion mutations.

## Author Contributions

Rui Meng conceived the study. Mingyuan Du and Leichong Chen collected the literature and drafted the manuscript. Cuiwei Liu and Zhenyu Li participated in collecting data and revised the manuscript. Sijia Zhang prepared the figures and participated in writing. All authors read and approved the final manuscript.

## Consent

The patients' information were anonymized and unidentified. The written‐informed consents have been provided by the patients to have the case details and any accompanying images published.

## Conflicts of Interest

The authors declare no conflicts of interest.

## Data Availability

The data analyzed in this study are available from the authors on reasonable request.
